# Designing noninferiority tuberculosis treatment trials: Identifying practical advantages for drug regimens with acceptable effectiveness

**DOI:** 10.1371/journal.pmed.1002850

**Published:** 2019-07-12

**Authors:** Piero L. Olliaro, Michel Vaillant

**Affiliations:** 1 Centre for Tropical Medicine and Global Health, Nuffield Department of Medicine, University of Oxford, Oxford, United Kingdom; 2 Competence Center in Methodology and Statistics, Department of Population Health, Luxembourg Institute of Health, Strassen, Luxembourg

## Abstract

In this Collection Review for the Novel Treatments for Tuberculosis Collection, Piero Olliaro and Michael Vaillant discuss the considerations when choosing a non-inferiority margin that is meaningful from statistical, ethical, clinical, and health standpoint.

**Summary points**

- The noninferiority design is being adopted in tuberculosis treatment trials to identify regimens that may have practical advantages over current standard therapy (e.g., being shorter, easier to adhere) and thus are more efficient in real-life settings, even while accepting that they might be less effective to a certain degree.
- This margin of acceptance is called the noninferiority margin, or delta. How narrow or wide the margin should be, and how this translates into acceptable losses and desired gains, is a matter of debate.
- Noninferiority trials are trials of ‘trade-offs’, in which one has to decide what one can lose in terms of pure efficacy against what one expects to make up in terms of effectiveness, tolerability, deployability, affordability, or else when replacing an existing intervention with a new one.
- This paper is about the principles behind identifying a ‘meaningful noninferiority margin’—that is, a margin that is meaningful from a statistical, ethical, clinical, and health standpoint.
- Pragmatic approaches to expressing treatment effects using the number needed to treat (NNT), the reciprocal of the absolute risk reduction, with NNT for one patient to benefit (NNTB) and NNT for one patient to be harmed (NNTH) are useful to understand the implications of outcome definition and find a way to quantify gains and losses.
- Applying the noninferiority design to pragmatic (effectiveness) trials in addition to efficacy/safety trials would help quantify the trade-offs in real life.

## Introduction

Identifying effective regimens for tuberculosis (TB) is challenging; trials are long between treatment and follow-up and require large sample sizes, so they take a long time to complete and are expensive. Oftentimes, they are also inconclusive. Lienhardt and Nahid [1] and Phillips and colleagues [2] call for innovation in trial design that would allow for identifying effective regimens more quickly and efficiently.

Hardly present in the medical literature before the year 2000, the noninferiority design has gained in popularity across disciplines and medical interventions in the past 2 decades. A recent paper [3] and the ensuing debate it generated [4–7] illustrate some of the controversies regarding this approach. An extension of the Consolidated Standards of Reporting Trials (CONSORT) statement covers the reporting of noninferiority trials [8], and there is regulatory guidance on the design of noninferiority trials [9, 10].

The noninferiority design is generally chosen when it is felt that a new medicine or intervention conveys benefits over the existing approved standard of care (such as better tolerability, real-life effectiveness, accessibility, or affordability), which would be enough to justify a ‘trade-off’ [4] between these advantages and an ‘acceptable’ loss of efficacy. A change in practice would be warranted if the new intervention is as effective or better (superiority would be preferred) but not if it is worse than the standard of care by a predefined noninferiority margin (also known as delta) [8]. The challenge with this design is 2-fold: (1) to identify an appropriate noninferiority margin so as to avoid retaining a harmful treatment because it has wrongly been judged noninferior [11], but also inappropriately discarding a treatment that brings a true benefit for the patient [12], and (2) to quantify how gains may offset losses.

Central to the design of these trials is therefore establishing a noninferiority margin, which should ‘preserve a minimum clinically acceptable proportion of the effect of the active treatment compared with placebo. This margin cannot be greater than the smallest effect size for the active treatment that would be expected in a placebo-controlled trial’ [13]. However, the delta should be ‘meaningful’ not just in statistical terms but also for patients and health systems on clinical, ethical [14, 15], and public health grounds.

**Fig 1:**
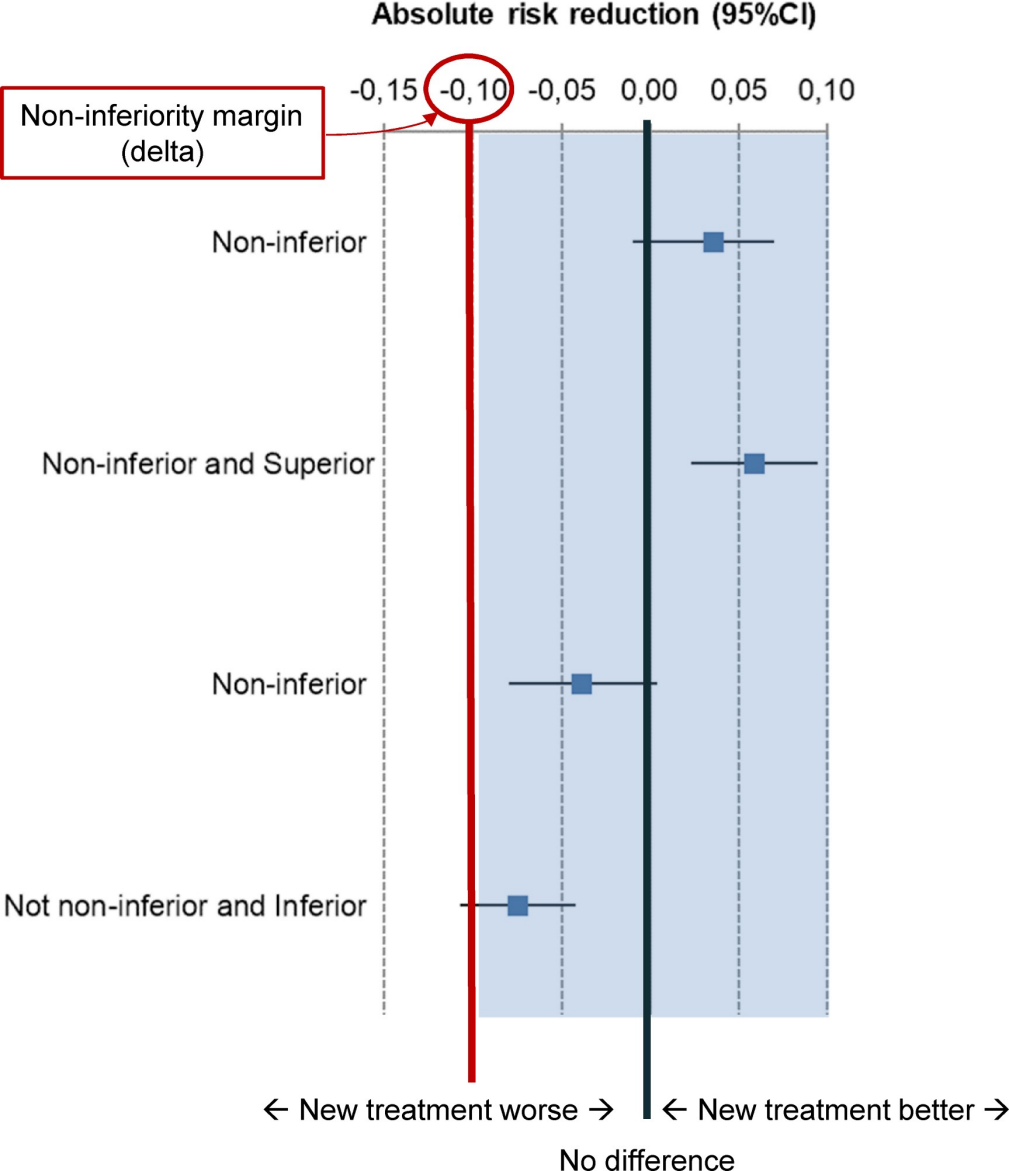
Interpretation of treatment differences in noninferiority trials comparing unfavourable outcomes with a new intervention versus active control. Error bars are 95% CI of treatment differences expressed as ARR. Arbitrary values are used for illustration purposes. Outcomes on the right of the ‘zero’ (no-difference) line favour the new treatment. Noninferiority margin set at −10%. ARR, absolute risk reduction; CI, confidence interval.

With the noninferiority design, the null hypothesis is that treatments are different, a type I error is to wrongly accept an inferior intervention, and a type II error is to reject a noninferior intervention [8]. The statistical procedure to test noninferiority is typically a one-sided test with a 97.5% level of significance or, preferably, a two-sided test with a 95% level of significance [3, 8]. When the treatment outcome is binary (e.g., success or failure), regimens are compared by calculating either a relative risk (RR), an odds ratio (OR), or an absolute risk reduction (ARR, also known as risk difference) and then calculating the (crude or adjusted) difference in failure (or success) rates between test and control treatment and the confidence interval (CI) around it. In order for a new treatment to be deemed noninferior to the comparator standard treatment, the lower bound of the CI (in the case of the risk difference between failure rates between control and test treatment) must be within the noninferiority margin (see Fig 1).

Although opinions have shifted over the years, it is now generally agreed that conclusions should take into account the result of the analyses of both the modified intent-to-treat (mITT) and the per-protocol (PP) population and that conclusions are more robust when the results of the analyses of both sets are consistent [8, 16]. The sample size of a noninferiority trial will depend on how narrow or wide the noninferiority margin is, the level of confidence, and the power chosen.

In this paper, we consider the implications of the noninferiority design for TB treatment trials, identify specific issues, and propose practical options. In particular, we focus on the choice of the noninferiority margin and clinically relevant end points; how these can be taken into account to weigh losses versus gains; and how to link statistical, clinical, ethics, patients’, and public health imperatives in a way that these studies can be designed and interpreted with a view to informing policy decisions and ultimately improving health outcomes.

## Noninferiority design in TB treatment trials

The noninferiority design has been adopted in explanatory treatment trials of active TB for newly diagnosed (expectedly drug-sensitive) TB (DSTB) (five trials completed and reported [17–21] and one systematic review [22]) and three for drug-resistant TB [DRTB] [23–25]. In these trials, the noninferiority margin ranged from 4% to 12% and is wider for multidrug-resistant TB (MDRTB) than DSTB (see Table 1).

**Table 1:** Overview of TB noninferiority treatment trials.

Indication	Study [Reference]	Regimen	Comparator	Delta	Outcome	Note
DSTB	Jindani, 2004 [19]	8HRZE	6HRZE	5%	inferior	
DSTB	Jindani, 2004	8HRZE (weekly*)	6HRZE	5%	inferior	
DSTB	Gillespie, 2014 [17]	4HRZM	6HRZE	6%	inferior	
DSTB	Gillespie, 2014	4RZEM	6HRZE	6%	inferior	
DSTB	Jindani, 2014 [18]	4HRZM	6HRZE	6%	inferior	
DSTB	Jindani, 2014	6HRZM	6HRZE	6%	noninferior	
DSTB	Merle, 2014 [21]	4HRZG	6HRZE	6%	inferior	
DSTB _noncavitary	Johnson, 2009 [20]	4HRZE	6HRZE	5%	inferior	
DSTB noncavitary	Alipanah, 2016 [22]	4HRZM/E	6HRZE	6%	noninferior	meta-analysis
DSTB	Lienhardt, 2011 [24]	6HRZE_fixed	6HRZE_loose	4%	noninferior	
DSTB	Aseffa, 2016 [23]	6HRZE fixed	6HRZE_loose	4%	noninferior	
DSTB	TBTC Study 31	4RHE; 4RptZHE	6HRZE	6.6%	enrolling	
DSTB + DRTB	STAND	PaMZ	6HRZE	12%	active, nonrecruiting	NCT02342886
MDRTB	STREAM [25]	40–48 weeks	18–24 months	10%	noninferior	NCT02409290
MDRTB	endTB	5 arms	SOC	12%	enrolling	NCT02754765
MDRTB	PRACTECAL	2 arms	SOC	12%	enrolling	NCT02589782

* In the continuation phase.

Abbreviations: DRTB, drug-resistant TB; DSTB, drug-sensitive TB; E, ethambutol; G, gatifloxacin; H, isoniazid; M, moxifloxacin; MDRTB, multidrug-resistant TB; Pa, pretomanid; R, rifampicin; Rpt, rifapentine; SOC, standard of care; STAND, Shortening Treatments by Advancing Novel Drugs; TB, tuberculosis; TBTC, Tuberculosis Trials Consortium; Z, pyrazinamide.

The standard treatment for newly diagnosed DSTB is a 6 month regimen made of a 2 month, four-drug intensive phase with daily isoniazid (H), rifampicin (R), pyrazinamide (Z), and ethambutol (E), followed by a 4 month, two-drug phase with H and R (4HRZE/2HR) [26]. This regimen is generally very effective if adhered to but is usually less so in routine practice, in which compliance is lower than in trial conditions; its performance varies even across clinical studies, also depending on trial methodology [27], including the type of culture used (solid versus liquid media), the population analysed (PP versus mITT) and the efficacy end points adopted—the latter being particularly relevant here, and it will be further discussed in this paper.

The current standard WHO-recommended ‘conventional’ regimen for MDRTB requires 18–20 months [28] with an (up to) 8 month intensive phase with four or more second-line drugs followed by a 12 month (or more) continuation phase with three or more second-line drugs. A shorter regimen of 9–12 months may be used in patients with R-resistant TB or MDRTB who were not previously treated with second-line drugs and in whom resistance to fluoroquinolones and second-line injectable agents was excluded or is considered highly unlikely [29]. Patient retention with such long, cumbersome, and potentially toxic regimens is a major challenge [30].

Of the trials listed in Table 1, so far, noninferiority has been demonstrated in DSTB in the following cases: a 6 month fluoroquinolone-substitution regimen delivered intermittently in the continuation phase including rifapentin versus standard 6 month daily regimen [18] (noninferiority margin 6%); fixed-dose versus loose (separately formulated drugs) combination [23, 24] (noninferiority margin 4%); and a 4 month fluoroquinolone-substitution regimen (with either gatifloxacin or moxifloxacin) versus a standard 6 month regimen in a meta-analysis of noncavitary TB (noninferiority margin 6%) [22]. Of the studies in DRTB, a 9–11 month regimen proved noninferior to the standard 20 month regimen [25].

Nunn and colleagues [31] illustrate a procedure to adopt when estimating the margin of noninferiority and the issues related to using the PP or mITT populations and dealing with missing data. Critical in this calculation is the choice of the trial end point, which in Nunn and colleagues is the relapse rates, assuming an insignificant number of primary on-treatment failures. Nunn and colleagues expect the relapse with current standard regimen in trial conditions to be 5%, which is broadly consistent with the findings of a trial by Jindani and colleagues [19] and a Cochrane systematic review [32] in which the relapse rate of reference regimens given for 4.5–12 months was 3.2 (95% CI 2.5%–4%). Using early TB trial data, Nunn and colleagues concluded that, when shortening the treatment from 6 to 4 months (a one-third reduction in duration), the expected difference in relapse rate would be 9%–10% with the current standard regimen for DSTB.

Noninferiority trials of DSTB so far have used noninferiority margins ranging from 4% to 6.6% (mostly 6%) (Table 1) and, instead of relapse rates, a composite end point (‘unfavourable outcome’, including primary failure during treatment, relapse during follow-up, and death) [17–19, 21]. In these trials, the overall rate of unfavourable outcomes at an 18 month follow-up with the standard regimen ranged from about 13% [21] to 20% [20].

**Fig 2:**
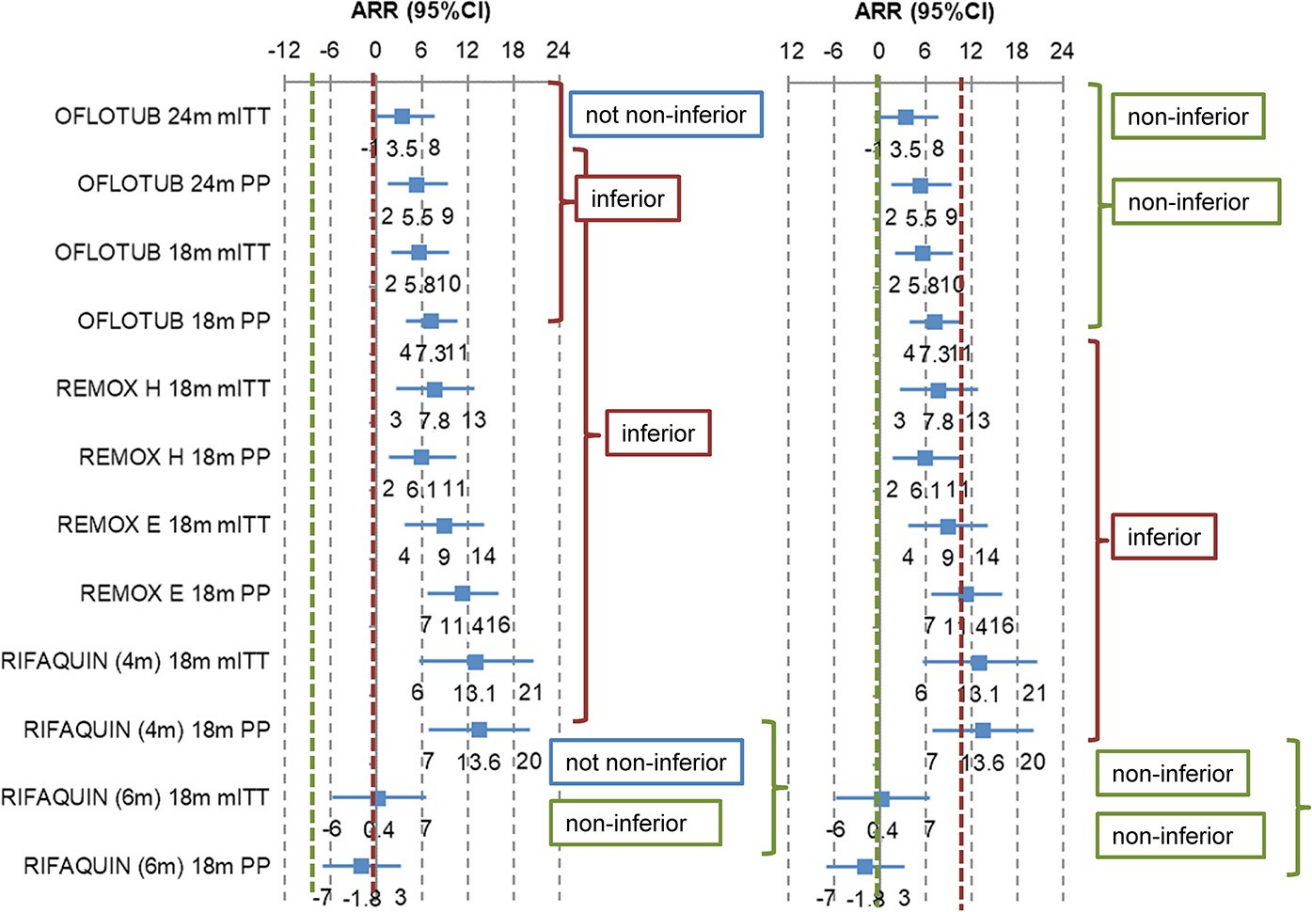
Unfavourable treatment outcome with fluoroquinolone-substitution regimens versus standard regimen for DSTB expressed as ARR with 95% CIs interpreted against a 6% (predetermined) and a 10% (post hoc) noninferiority margin. 18m, 18 month follow-up from treatment start; 24m, 24 month follow-up after treatment end; ARR, absolute risk reduction; CI, confidence interval; DSTB, drug-sensitive tuberculosis; mITT, modified intent-to-treat set; PP, per-protocol set.

A 4-percentage-point shift from a 6% to a 10% margin (see Fig 2) would mean that one of the fluroquinolone-substitution regimens would have been deemed noninferior had the larger delta been adopted [21].

## ‘Meaningful’ noninferiority margin

This example further illustrates how critical it is to establish a noninferiority margin that is ‘meaningful’ statistically, clinically, and programmatically and is ethically acceptable. But how can the results of a trial be made to speak to clinicians and policy makers?

We propose to translate the ARR into number needed to treat (NNT). Although objections have been raised to the use of NNTs [33, 34], and despite some statistical limitations, the NNT conveys a message that is easier for clinicians and policy makers to understand when it comes to quantifying the trade-offs between two interventions [35, 36]. We also support the use of the terms NNT for one patient to benefit (NNTB) and NNT for one patient to be harmed (NNTH) with the test regimen when compared with the control regimen, as proposed by Altman [37].

**Fig 3:**
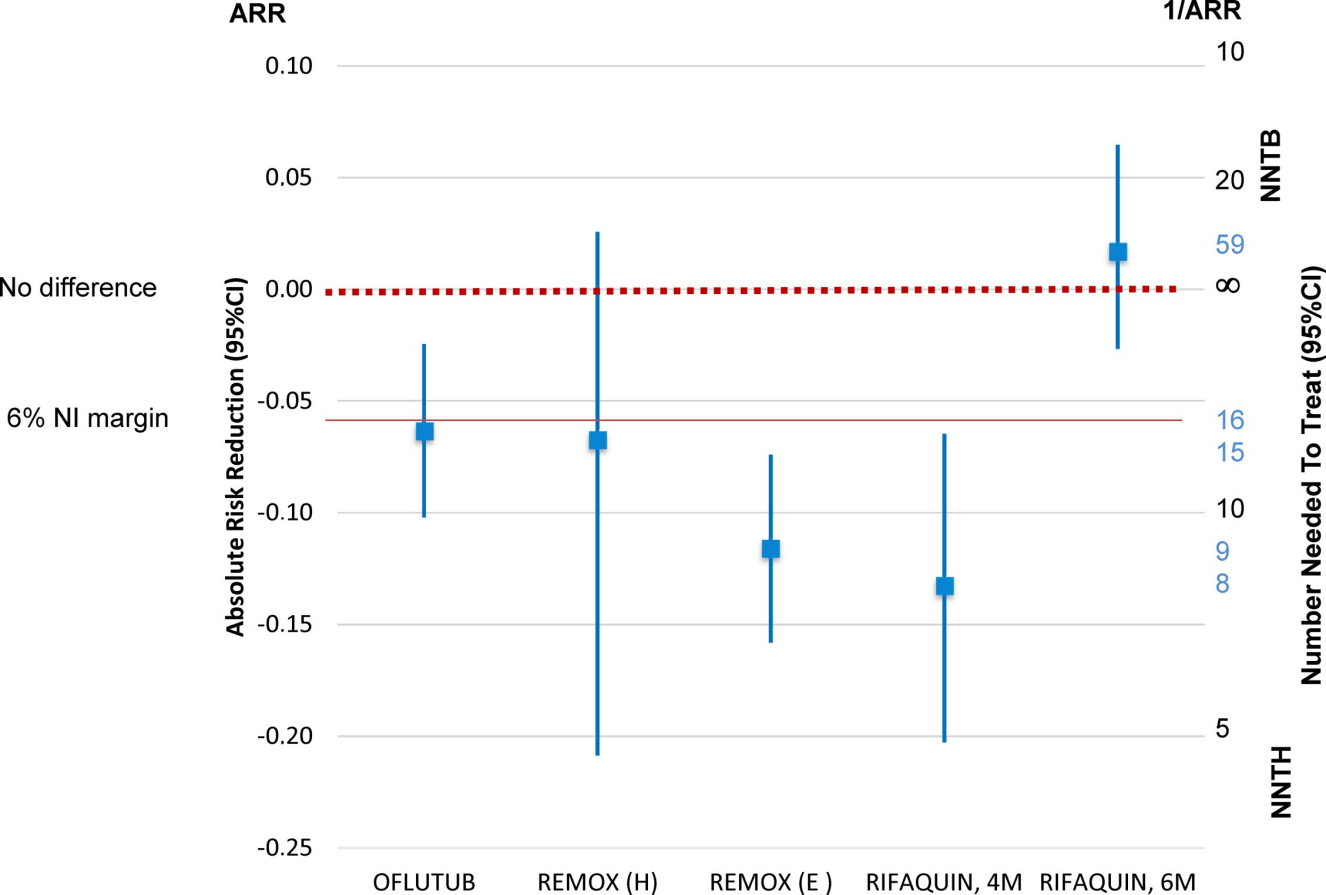
Fluoroquinolone-substitution trials in DSTB: 18 month rates of unfavourable outcome in the mITT population expressed as ARR between test versus control regimen and corresponding number needed to treat. ARR, absolute risk reduction; CI, confidence interval; DSTB, drug-sensitive tuberculosis; E, ethambutol; H, isoniazid; mITT, modified intent-to-treat; NI, noninferior; NNTB, number needed to treat for one patient to benefit; NNTH, number needed to treat for one patient to be harmed.

The NNT is easy to calculate: it is the reciprocal of the ARR (NNT = 1 / ARR); similarly, the CI is calculated by inverting and exchanging the upper limit (UL) and lower limit (LL) of the CI for ARR [1 / UL (ARR), 1 / LL (ARR)]. However, complications arise when there is no difference between treatments because, when the ARR is zero, the NNT is infinite, and the CI of the NNT will comprise infinity, thus violating the continuity between the CI limits. The classical Wald’s CIs suffers from a series of limitations (see, for instance, Newcombe [38]), and alternatives have been proposed, such as Cook and Sacket’s [39]—which we use for our calculations in this paper—Schultzer and Mancini’s [40], and Wilson scores [41]. We use here the NNT scale proposed by Altman [37] (Fig 3, Table 2).

**Table 2:** NNTs with Cook and Sacket’s 95% CIs based on 18 month unfavourable outcome with fluoroquinolone-substituted regimens versus standard TB regimen in the PP and mITT populations.

Clinical trial	PP	mITT
OFLOTUB	NNTH = 16 (41–10)	NNTH = 13 (24–9)
REMOX (H)	NNTH = 15 (NNTH 5 to ∞ to 39)	NNTH = 17 (NNTH 6 to ∞ to 47)
REMOX (E)	NNTH = 9 (14–6)	NNTH = 11 (16–8)
RIFAQUIN, 4M	NNTH = 8 (15–5)	NNTH = 9 (19–6)
RIFAQUIN, 6M	NNTB = 59 (NNTB 17 to ∞ to NNTH 40)	NNTB = 120 (NNTB 25 to ∞ to NNTH 42)

Abbreviations: CI, confidence interval; E, ethambutol; H, isoniazid; mITT, modified intent-to-treat population; NNT, number needed to treat; NNTB, NNT for one patient to benefit; NNTH, NNT for one patient to be harmed; PP, per-protocol; TB, tuberculosis.

Example 1: fluoroquinolone-substitution trials in DSTB. In the OFLOTUB trial [21], if we take the 18 month follow-up unfavourable outcome end point with the 4 month gatifloxacin-containing regimen versus standard treatment, the ARR (95% CI) is −6.4% (−10.2% to −2.4%) on the PP population analysis and −7.4% (−10.7% to −4.2%) on the mITT population—thus, this regimen is not noninferior and is inferior, respectively, to the standard regimen, as all confidence limits sit on one side of the no-difference line. This translates into an NNTH ranging from 41 to 9 between the two analysis populations, which means that a one-third reduction in treatment duration will cause one more patient to fail (compared with the standard 6 month regimen) between every 41 (best case) and 9 (worse case) patients treated.

By contrast, with a 6 month moxifloxacin regimen with rifapentin given intermittently in the continuation phase (the RIFAQUIN trial [18]), the confidence limits stretch across the no-difference line, and the NNT includes infinity (e.g., NNTB 25 to infinity to NNTH 42 in the mITT analysis). This means that with this regimen, an NNTB better than 25 is unlikely (i.e., that in order to obtain one more success over standard treatment, one would need to treat at least 25 participants). At the same time, an NNTH worse than 42 is also unlikely (i.e., that for one more patient to be harmed, at least 42 will have to be treated).

Example 2: STREAM trial in MDRTB [23]. This trial compared a shorter (9–11 month) regimen to the ‘classical’ 20 month regimen with a 10% noninferiority margin. The failure rates in the test and control arms in the mITT population (*n* = 253 and 130, respectively) were 21.2% versus 20.2%, with an unadjusted ARR for failure between control and test treatment of −1% (95% CI 7.5% to −9.5%). This translates into NNT −100 (NNTB 13 to infinity to NNTH 11), which means that an NNTB better than 13 and an NNTH worse than 11 are unlikely. Similar conclusions are derived from the PP set: ARR (95% CI) 0.7 (10.5 to −9.1) for NNT (95% CI) 143 (NNTB 7 to infinity to NNTH 11).

## Composite versus individual study end points

Using a composite end point is practical (as it summarises findings into a single message), but we must be aware of two potential issues.

**Fig 4:**
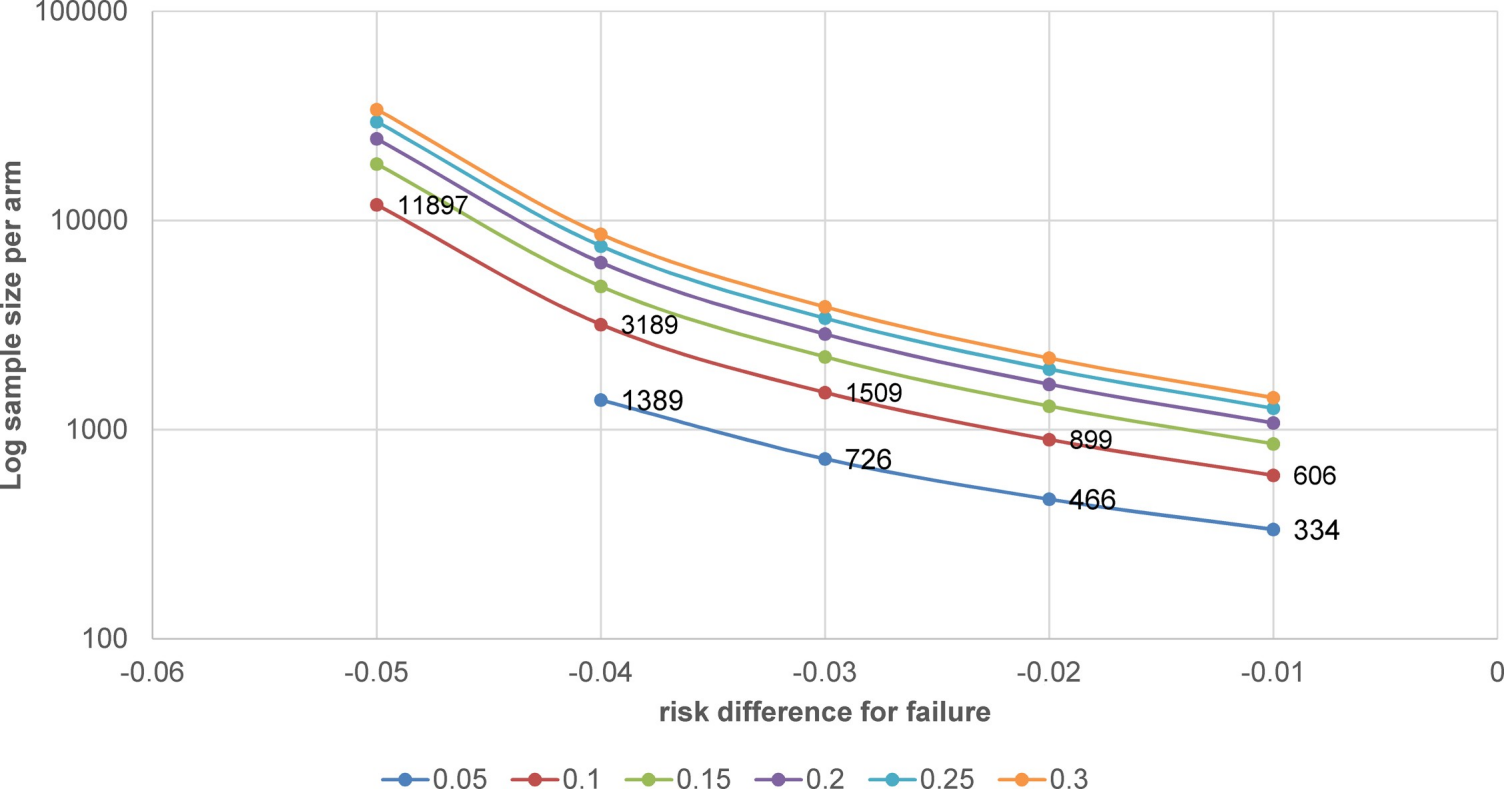
Example of sample size calculation with 1:1 allocation for noninferiority trial with noninferiority margin = 6%, failure rates in the control arm ranging from 5% to 30% (solid lines), and risk difference for failure between control and test treatment ranging from 1% to 5% (x-axis). The sample size was calculated with a one-sided chi-squared test for comparison of two groups by specifying the delta, the reference proportion, and the expected difference. The test statistic is assumed to have a null distribution of N(0,1). A description of the underlying calculations can be found in Julious and Owen [42].

One is that, as mentioned earlier, changing from ‘relapse’ to ‘unfavourable outcome’ (generally including primary failure, relapse, and death) inflates the failure rate and has effects on the power and sample size calculation of the study. For instance, a change from 5% to approximately 10%–15% failure rate (depending on the population analysed) means that the required sample size could double or triple; for a noninferiority margin set at 6%, the sample size would increase by 1.8%–2.6%, 1.9%–2.8%, 2.1%–3.1%, and 2.3%–3.5% for risk differences from 1% to 4%, respectively. An example of implications for sample size calculation is presented in Fig 4.

**Fig 5:**
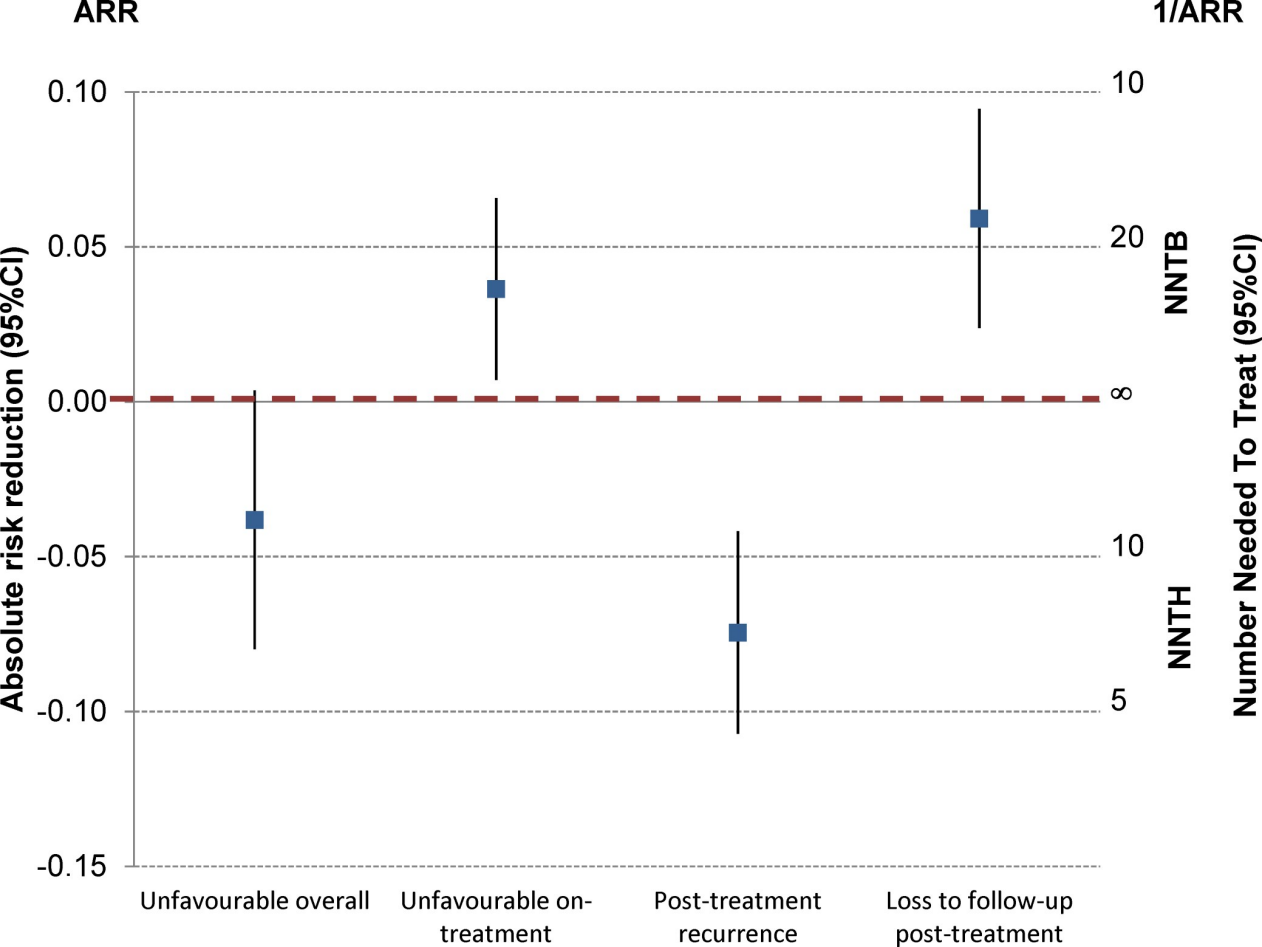
Composite outcome versus on- and posttreatment outcomes—OFLOTUB trial [21]. ARR, absolute risk reduction; CI, confidence interval; NNTB, number needed to treat for one patient to benefit; NNTH, number needed to treat for one patient to be harmed.

The other complication with a composite end point is that there might be discordant results within its individual components. An illustrative example can be found in the OFLOTUB trial (Fig 5) [21].

The primary efficacy end point was a composite unfavourable outcome including on-treatment events (failure, death, other adverse event, drop-out, and withdrawal) plus posttreatment recurrence by month 24 on the mITT population. This gives an unadjusted ARR (95% CI) of −3.8% (0.4% to −8%). However, when one looks at these two components separately, the short regimen is significantly better than the standard treatment for on-treatment outcomes (ARR [95% CI] 3.6% [0.7%–6.6%]) but significantly worse for relapse (−7.5% [−4.2% to −10.7%]); at the same time, it has also significantly fewer posttreatment losses to follow-up (5.9% [2.4%–9.5%]).

It is therefore prudent to dissect composite end points in order to verify that the individual components do not have conflicting implications for regimen effectiveness [3]. Analysing the granularity of the results is important also for practical reasons. We must know what we have to watch out for when it comes to decide what type of losses we are prepared to accept in terms of efficacy. Type and timing of failure is of paramount importance. Patient retention is a challenge, especially after treatment is completed; long-term posttreatment follow-up is required in TB to make sure the patient does not relapse. For instance, in Jindani and colleagues [19], in the 6 month standard therapy group, there were three times as many patients lost because they did not report to a posttreatment follow-up visit as those not reporting while on treatment for DSTB (12% versus 4%). Primary (on-treatment) failures are easier to detect, especially in clinical trials and in routine practice when treatment is supervised; posttreatment relapses may be more challenging, as patients are generally less compliant with follow-up visits, at least for as long as they are not unwell.

## Trading gains for losses

Now the question is, Using the previously mentioned examples, how would stakeholders (national TB programme managers, caregivers, patients) weigh losses and gains?

In the case of a one-third reduction of treatment for DSTB given under directly observed treatments (DOTs), what would be the practical gains for the health system (e.g., more time for patient visits, reduced costs, increased efficiency) versus having to deal with one more failure every 10 rather than 40 cases? Would the advantages of a shortened treatment and faster resolution, along with the smaller reduction in wages for the patients, outweigh the disadvantages of excess relapses? Can a national TB programme gear up for actively and systematically following up with patients in order to identify and deal with relapses promptly?

Another example for DSTB: How would health providers and patients value a regimen that is given for the same total duration but weekly (instead of daily) in the 4 month continuation phase [18] when this regimen is estimated to be producing a benefit every approximately 20 treatments or one more failure every approximately 40?

Similarly, for MDRTB, when a patient is now on treatment for 1.5–2 years, how would they value a regimen that is half as long and might either produce a benefit every 7–13 patients treated or one more failure every 11? The gains here may, however, be offset by the need for drug sensitivity testing and by the toxicity of injectable aminoglycosides used in these shorter regimens, at least until evidence is gained on replacing them with safer drugs [28], or by a higher risk of selecting for drug-resistant bacteria.

**Fig 6:**
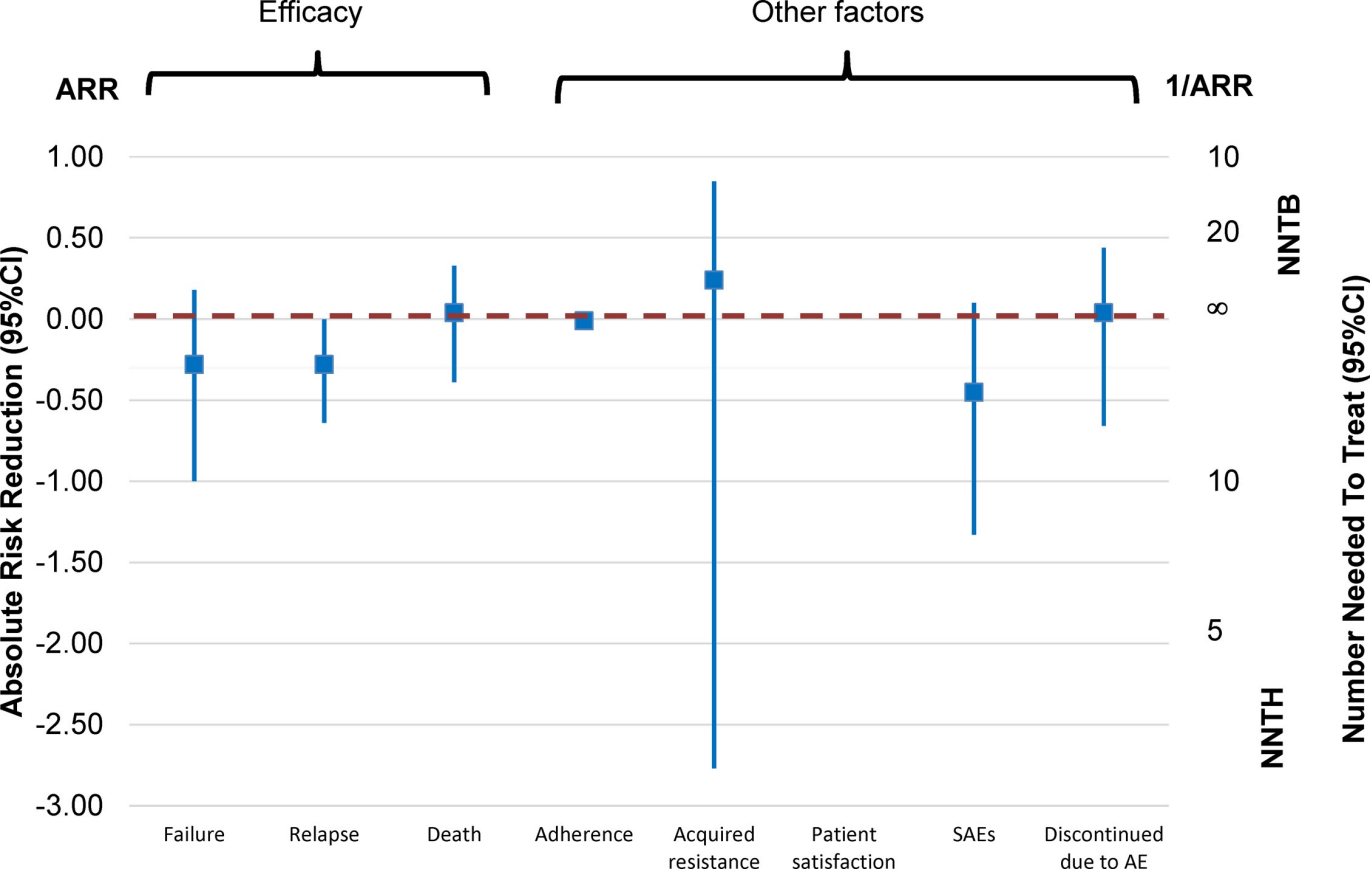
Plotting efficacy outcomes and examples of other relevant factors contributing to weigh gains and losses—Cochrane systematic review and meta-analysis of fixed-dose versus loose combinations [43]. AE, adverse event; ARR, absolute risk reduction; CI, confidence interval; NNTB, number needed to treat for one patient to benefit; NNTH, number needed to treat for one patient to be harmed; SAE, serious adverse event.

Where would one draw the line? Information is required on a number of variables which, together, can help quantify gains and losses and thus inform both study design and treatment policy decisions. These cover a range of outcomes—not just efficacy and safety but also patient’s preferences and satisfaction, quality of life, healthcare provider’s preferences and performance, emergence of drug resistance, etc., which are rarely collected in clinical trials. Fig 6 (derived from the Cochrane systematic review of fixed-dose versus loose combination treatment [43]) offers an example of some of the criteria which could be used to compare gains and losses (limited information was collected on patient’s satisfaction, so this could not be plotted).

Together with key stakeholders, we need to identify the critical questions and score the answers in order to inform both study design (and identify a ‘meaningful noninferiority margin’ in case of a noninferiority trial) and policy and practice (how to take advantage of the benefits and how to handle the potential problems).

Traditionally, we tend to derive this information from explanatory (‘phase 3’) trials. However, they suffer from an inherent limitation in this regard, in that they typically seek to measure the efficacy (and safety) gains introduced by a new intervention by minimising confounders and standardising eligibility criteria—the very elements we are interested in to decide on the trade-offs. Instead, in order better to quantify gains and losses of a new treatment and its associated effects, it would be useful to apply the noninferiority design also to pragmatic (effectiveness) trials, analysed on the (m)ITT (as well as PP) population.

## Conclusions

Though it has been wrongly used to justify ‘me-too’ medicines, the noninferiority design is having a growing place in diseases like TB, which require treatments that are long and cumbersome for patients and health systems alike, and where attributes like adherence, user-friendliness, and tolerability are critical to real-life effectiveness. Although the noninferiority design may be applied to treatment trials in both DSTB and DRTB against the current standard of care, this does not take away the responsibility for finding both more effective and easier-to-comply regimens, especially for DRTB.

This design responds to the need for a planned trade-off between what we think we can afford to lose in terms of efficacy against what we expect to gain in terms of safety, effectiveness, ease of use, costs, etc. It is generally applied when a net gain in efficacy cannot realistically be shown within the conditions of a typical trial, though a superiority test can be applied if noninferiority is demonstrated. However, more work is required to develop end points for TB treatment trials, which will identify regimens that better serve the needs of patients as well as country TB programmes and health providers. Also, using the noninferiority design in pragmatic trials would provide useful information.

The noninferiority margin is a central element in study design and interpretation. Identifying and weighing the appropriate parameters for gains and losses is crucial towards defining a ‘meaningful’ noninferiority margin.

## Abbreviations

ARR: absolute risk reduction
CI: confidence interval
CONSORT: Consolidated Standards of Reporting Trials
DOT: directly observed treatment
DRTB: drug-resistant TB
DSTB: drug-sensitive TB
E: ethambutol
H: isoniazid
LL: lower limit
MDRTB: multidrug-resistant TB
mITT: modified intent-to-treat
NNT: number needed to treat
NNTB: NNT for one patient to benefit
NNTH: NNT for one patient to be harmed
OR: odds ratio
Pa: pretomanid
PP: per-protocol
R: rifampicin
RR: relative risk
STAND: Shortening Treatments by Advancing Novel Drugs
TB: tuberculosis
TBTC: Tuberculosis Trials Consortium
UL: upper limit
Z: pyrazinamide.
